# Continuous Cropping of Patchouli Alleviate Soil Properties, Enzyme Activities, and Bacterial Community Structures

**DOI:** 10.3390/plants13243481

**Published:** 2024-12-12

**Authors:** Muhammad Zeeshan Ul Haq, Guangtao Gu, Ya Liu, Dongmei Yang, Huageng Yang, Jing Yu, Yougen Wu

**Affiliations:** School of Breeding and Multiplication (Sanya Institute of Breeding and Multiplication), School of Tropical Agriculture and Forestry, Hainan University, Sanya 572025, China; drzeeshanulhaq@gmail.com (M.Z.U.H.);

**Keywords:** continuous cropping obstacles, bacterial community, *Pogostemon cablin*, soil properties, soil enzyme activities

## Abstract

*Pogostemon cablin* (Patchouli), an essential medicinal plant in the *Lamiaceae* family, faces significant challenges under continuous cropping (CC) obstacles. This study examined the rhizospheric soil bacterial communities of patchouli under four different CC years, zero (CK), one (T1), two (T2), and three (T3) years through high-throughput 16S rRNA gene amplicon sequencing. Results showed long-term CC led to significant soil properties and enzyme activity shifts. Key parameters such as soil pH and total potassium (TK) decreased, while ammonium nitrogen (NH_4_^+^–N), soil organic carbon (SOC), nitrate nitrogen (NO_3_^−^–N), available potassium (AK), available phosphorus (AP), total nitrogen (TN), and total phosphorus (TP) increased over the cropping years. Enzyme activities, including ß-glucosidase (ß-GC), polyphenol oxidase (PPO), catalase (CAT), N-acetyl-β-D-glucosaminidase (NAG), and leucine aminopeptidase (LAP), were notably affected. The CC altered the bacterial community structure and composition, reducing the relative abundance of Proteobacteria, Firmicutes, Actinobacteria, and Planctomycetota over time. These findings highlight the impact of CC on patchouli rhizosphere bacteria, providing insights for improved soil management and fertilization strategies in CC systems.

## 1. Introduction

*Pogostemon cablin* (Patchouli), a medicinal plant native to Southeast Asia, is mainly cultivated in southern China, especially in the Guangdong and Hainan provinces [1]. Patchouli is categorized into four types (Nanxiang, Paixiang, Zhaoxiang, Zhanxiang) in traditional Chinese medicine, where its dried parts (aboveground) are used to treat fever, nausea, headaches, and indigestion, and for their anti-inflammatory and analgesic effects [2]. Additionally, patchouli volatile oil, extracted from its leaves, contains significant amounts of ketones and alcohol, essential for enhancing fragrance longevity in the perfume industry [3].

Despite its esteemed status as a medicinal plant, patchouli faces difficulties regarding continuous cropping (CC) obstacles. CC refers to the repeated cultivation of the same crop or closely related species on the same land, often leading to declines in crop growth, yield, and quality [4]. These challenges are attributed to various factors, including the accumulation of soil-borne pathogens, soil degradation, and nutrient depletion, which collectively compromise plant health and productivity [5]. In China, CC exerts pressure on the soil, causing soil infertility, ecosystem degradation, and reduced productivity. Early detection of soil health decline is key to sustainable management [6]. Recent studies indicated that CC deteriorates rhizosphere soil, disrupts microbial communities, stunts plant growth, raises disease incidence, and can lead to autotoxicity, soil infertility, and plant death [7,8]. In total, 70% of the medicinal plants, including *Lepidium meyeni*, *Rehmannia glutinosa*, *Panax ginseng*, *Angelica sinensis*, *Panax quinquefolius*, *Panax notoginseng*, *Andrographis paniculate*, and *Condopsis tangshen*, whose roots or rhizomes are used for medicinal purposes, face varying degrees of CC challenges [4]. Similarly, this issue has become widespread in patchouli plant production, leading to severe soil-borne diseases, and reduced crop yields and quality, and hindering the industry’s sustainable development [9]. To address this, most commercial farmers alternate crops or leave fields fallow, though this reduces patchouli yields [10]. Some also increase fertilizer and pesticide use, causing soil contamination from chemical overuse [11].

The soil composition and diversity of bacteria are crucial for maintaining soil health. Soil microbes are crucial for element cycling and ecosystem functions, and their imbalance can signal human activity [12]. Healthy soil is crucial for plant growth and agricultural food security [13]. Given their key role in soil functions, microorganisms must be factored in when studying how agricultural soils respond to CC [14,15]. Studies have shown a link between soil enzyme activity and microbial composition in response to CC [16,17]. A higher abundance of bacteria and fungi positively correlates with soil ecosystem quality [18]. However, CC negatively affects soil microorganisms, reducing functional strains like aerobic and nitrogen-fixing bacteria and disrupting microbial communities, which hinders plant growth [19,20]. It also inhibits antibiotic secretion by beneficial bacteria and promotes pathogenic bacteria, increasing plant disease [21]. Additionally, harmful microorganisms secrete metabolites that attract beneficial ones, further disrupting soil microecology [22]. Therefore, studying the CC of patchouli’s effects on soil microbes and biochemistry is vital for improving patchouli practices and soil health.

Soil properties and enzyme activity are key indicators of soil ecosystem function, produced by soil fauna, microorganisms, and plant roots. Enzymes, as catalytic proteins, significantly accelerate biological reactions and are essential in the soil carbon (C), nitrogen (N), and phosphorus (P) cycles [23,24]. These reflect soil biochemical processes’ intensity, making them more sensitive than physicochemical changes. Thus, monitoring these activities is a more reliable way to assess the impact of CC on soil quality [25]. Recent studies on the effects of CC on enzymes show mixed results; some report a peak with increased planting years, while others find a negative correlation, possibly due to differences in soil type and plant species [26,27]. Additionally, microbial communities influence enzyme activities and maintain plant root microecology. While numerous studies have examined the impact of CC on patchouli plants’ roots, shoots, and their damage mechanisms, research on its influence on the rhizosphere microbial community structure and soil physicochemical properties remains limited [2,9]. This study aims to investigate the rhizosphere soil characteristics and bacterial community changes of patchouli under CC conditions. The specific objectives include the following: (1) examining the effects of CC on the chemical properties and enzyme activities of patchouli soil; (2) elucidating the interactions between soil properties, enzyme activities, and microorganisms; and (3) assessing the changes in microbial community diversity and structure under CC. Based on the findings of this research, future efforts can focus on developing more refined soil management strategies tailored to the variations in soil properties and bacterial communities associated with different durations of CC. These strategies may involve optimizing fertilization practices, improving soil structure, or introducing beneficial microorganisms to mitigate the adverse effects of CC and promote the sustainable development of patchouli cultivation.

## 2. Results

### 2.1. Impact of CC on Soil Chemical Properties

The soil chemical properties of the treatments, one-year CC soil of patchouli (T1), two-year CC soil of patchouli (T2), and three-year CC soil of patchouli (T3), showed significant changes compared to zero-year CC soil of patchouli (CK). The pH slightly decreased across all treatments, with a reduction of 0.97% in T1 and 1.58% in T2, and the most pronounced decline observed in T3, at 4.38% (Table 1). The soil organic carbon (SOC) showed significant increases, especially in T3, which exhibited a 370.11% increase, followed by T2 (312.07%) and T1 (264.37%), reflecting enhanced carbon sequestration. The ammonium nitrogen (NH_4_^+^–N) showed a slight increase in T1 (24.86%), remained stable in T2 (1.25%), and slightly declined in T3 (1.14%). Similarly, nitrate nitrogen (NO_3_^−^–N) showed a moderate increase in T1 (9.08%) and T2 (8.21%), while T3 decreased (18.10%). The most significant changes observed in available phosphorus (AP) exhibited a remarkable increase, especially in T3 (443.01%), with T1 (293.48%) and T2 (337.33%). Available potassium (AK) increased in all treatments, with T2 showing the highest change (211.02%), followed by T3 (85.38%) and T1 (43.83%). In contrast, total potassium (TK) decreased consistently across treatments, with 7.79%, 10.39%, and 15.58% reductions in T1, T2, and T3, respectively. Total nitrogen (TN) and total phosphorus (TP) exhibited significant increases, with TN rising by 245.83% in T1, 295.83% in T2, and 504.17% in T3, and TP increasing by 428.57% in T1, 362.86% in T2, and 337.14% in T3.

### 2.2. Impact of CC on Soil Enzyme Activities

**Figure 1:**
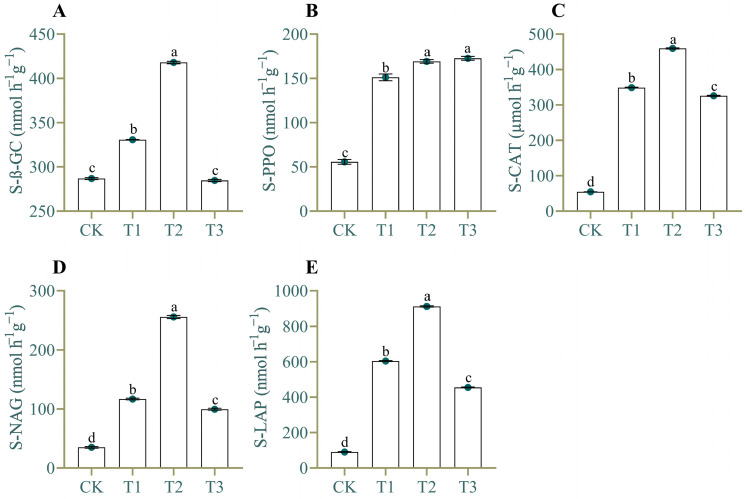
Impact of patchouli CC on soil enzyme activities. (**A**) The ß-Glucosidase (β-GC) activity, (**B**) polyphenol oxidase (PPO) activity, (**C**) catalase (CAT) activity, (**D**) N-acetyl-β-D-glucosaminidase (NAG) activity, and (**E**) Leucine aminopeptidase (LAP) activity determind under different CC years patchouli soil. Significant variations among the four “treatments” are signaled by unique small letter alphabets (*p* < 0.05) following Tukey’s HSD tests.

Similarly, different CC years of patchouli significantly affected the five enzyme activities (Figure 1). The results demonstrated notable enzyme activity variations across treatments (T1, T2, and T3) compared to the CK. The ß-Glucosidase (β-GC) activity increased in T1 and T2 by 15.34% and 45.74%, respectively, while T3 slightly decreased (−0.74%) compared to CK (Figure 1A). Similarly, polyphenol oxidase (PPO) activity increased across all treatments, with T3 leading at 209.68%, followed by T2 (203.23%) and T1 (170.97%) (Figure 1B). The catalase (CAT) activity showed a significant increase in T2 (741.32%), followed by T1 (538.52%) and T3 (496.18%) compared to CK (Figure 1C). The N-acetyl-β-D-glucosaminidase (NAG) peaked in T2 (630.51%), while T1 (233.63%) and T3 (184.51%) showed a more moderate increase (Figure 1D). Leucine aminopeptidase (LAP) activity was highest in T2 (907.48%), followed by T1 (567.69%) and T3 (402.47%) (Figure 1E). Overall, T2 showed the most significant enhancement of enzymatic activities, followed by T1, with T3 showing moderate but significant effects.

### 2.3. Impact of CC on Soil Bacterial Diversity and Structure

**Figure 2:**
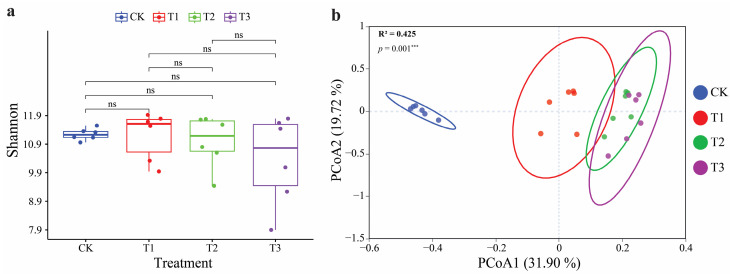
Impact of patchouli CC on the diversity of soil bacteria. The Shannon bacterial diversity in rhizosphere soil of the patchouli plant (**a**). The Bray–Curtis distance-based principal coordinate analysis (PCoA) for variations in the bacterial communities’ composition in the rhizosphere soil of patchouli in different CC years (**b**). The significance differences represented by *** *p* < 0.001. ns: not significant.

This study obtained 1,651,831 high-quality 16S rRNA sequences, identifying 143,708 bacterial ASVs (Table S1). No significant variations were observed in the Shannon index of bacteria across all treatments of patchouli CC rhizosphere soil (Figure 2a). Similarly, no significant differences were observed in the Simpson, Chao, Pielou, and Pd indices of bacteria across all treatments (Figure S1). Meanwhile, PCoA analysis was employed to analyze bacterial communities’ beta (ß) diversity in patchouli in the CC year soil and showed significant changes between different CC years of patchouli soil (Figure 2b). This indicated that CC has a prominent influence on bacterial communities’ rhizosphere soil of patchouli (*p* = 0.001 ***).

**Figure 3:**
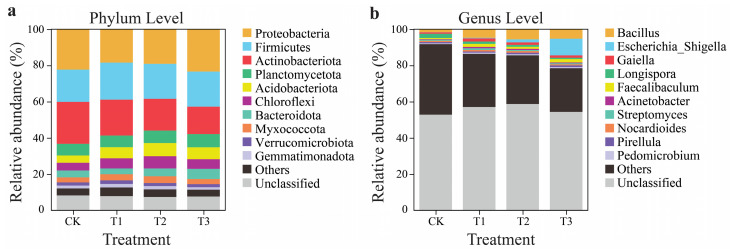
Soil bacterial communities’ relative abundance (%) at the genus and phylum levels. Variations in the relative abundance of bacterial phyla in the rhizosphere soil of patchouli during various CC years (**a**). Variations in the relative abundance of the bacterial genus in the rhizosphere soil of patchouli during different CC years (**b**).

The predominant bacterial phyla in all soil samples, exhibiting relative abundances over 5%, were Proteobacteria (19.12–23.47%), Firmicutes (17.76–20.40%), Actinobacteriota (15.08–23.22%), Planctomycetota (6.44–7.29%), and unclassified (7.34–8.18%). Additionally, the relative abundance in CK was better than that of the CC years’ soil of the patchouli plant at phylum levels (Figure 3a). Proteobacteria, a dominant phylum, decreased in T1 (17.26%) and T2 (14.54%) but showed a minor increase in T3 (4.87%). In contrast, Firmicutes exhibited a consistent increase in all treatments, with T1 showing the most significant rise (14.85%), followed by T3 (9.14%) and T2 (8.72%). Actinobacteriota, another key phylum, declined in all treatments, most significantly in T3 (35.06%). Planctomycetota remained relatively stable in T1, with slight increases in T2 (7.19%) and T3 (13.32%). Acidobacteriota and Chloroflexi increased significantly in all treatments, with T2 showing the highest increases for both phyla (83.11% and 58.47%, respectively). Bacteroidota also increased notably in T2 (11.87%) and T3 (50.86%), though it slightly decreased in T1. Myxococcota increased across most treatments, particularly in T2 (34.93%). However, Verrucomicrobiota and Gemmatimonadota displayed more moderate changes, with a minor increase in T1 and T2 but a slight decline in T3. Unclassified bacteria decreased across all treatments, with the most significant reduction in T2 (10.34%) (Table S2).

The microbial genus composition showed significant changes across the treatments (T1, T2, T3) compared to CK (Figure 3b). Bacillus increased significantly in all treatments, with the most considerable increase observed in T2 (220.17%), followed by T3 (201.62%) and T1 (171.79%). Escherichia significantly increased, especially in T3 (11,886.21%) and T2 (2,045.06%). Gaiella increased across treatments, particularly in T1 (126.06%), while T2 and T3 slightly increased (104.33% and 85.68%, respectively). Conversely, Longispora declined in all treatments, with the most significant drop in T3 (68.55%), followed by T2 (57.76%) and T1 (44.49%). Faecalibacterium showed a significant increase in T3 (207.77%) and T1 (182.32%) but a modest increase in T2 (85.18%). Acinetobacter also showed a significant increase in T1 (236.01%) but decreased in T2 (7.59%) and T3 (59.42%). Streptomyces showed moderate increases in T1 (45.29%) and minimal changes in T2 and T3, while Nocardiodes slightly increased in T1 (17.81%) and T2 (24.73%) but decreased in T3 (12.83%). Pirellula increased in T2 (17.97%) and T3 (28.57%) but decreased in T1 (14.64%). Pedomicrobium also slightly increased across all treatments, with T1 showing the highest increase (21.70%). The “Other” category, representing unfamiliar genera, decreased across all treatments, with T3 showing the most significant drop (37.82%). Similarly, the unclassified genera increased moderately, with T2 showing the most significant increase (11.13%) (Table S3).

**Figure 4:**
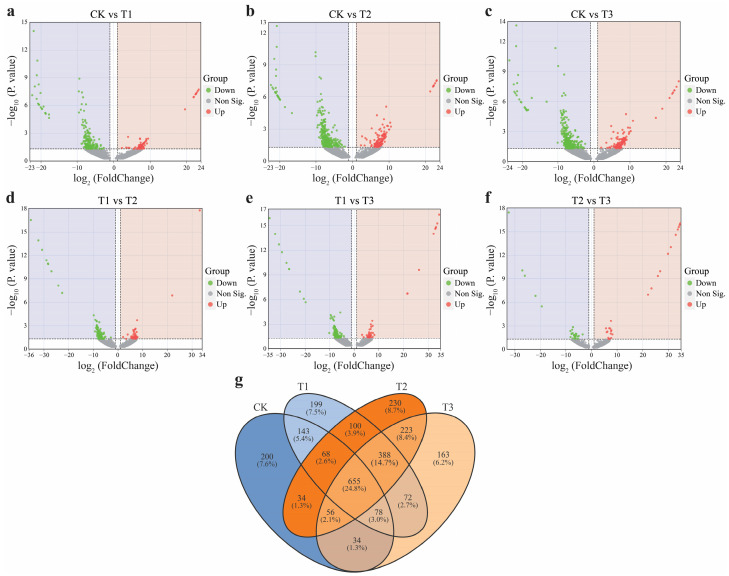
The CC years of patchouli plant soil result in enriched and depressed ASVs. A volcano plot was used to describe ASV differences in CK vs. T1 (**a**), CK vs. T2 (**b**), CK vs. T3 (**c**), T1 vs. T2 (**d**), T1 vs. T3 (**e**), and T2 vs. T3 (**f**) in the rhizosphere soil. The x-axis represents the log2 (FoldChange) between treatments, the y-axis represents the −log10 (*p* value), and each point represents a distinct ASV. Red dots indicate enriched ASVs, while the green denotes the depleted ASVs. Venn diagrams showed significantly enriched differential ASVs between different CC years of patchouli soil (**g**).

The alterations in bacterial species at the ASV level across different CC years in patchouli soil were analyzed. Our results revealed significant depletion and enrichment of ASVs in various comparisons. In the CK vs T1 comparison, the number of depleted ASVs was lower than that of enriched ASVs (Figure 4a). Similarly, the number of depleted ASVs was lower than that of enriched ASVs in the other comparisons (Figure 4b–f). To further explore the relationships among differential ASVs under varying CC years, the unique and shared enriched ASVs in patchouli rhizosphere soil were analyzed. In Figure (4e), the Venn diagram indicated that 655 ASVs were shared across the different CC years, highlighting their potential importance in supporting plant growth in patchouli soil under varying CC conditions. Additionally, numerous unique ASVs were identified in the CK, T1, T2, and T3 treatments, a total of 200, 199, 230, and 163 ASVs, respectively. The T2 treatment exhibited the highest proportion of unique ASVs, accounting for 8.7%, compared to 7.6% in CK, 7.5% in T1, and 6.2% in T3.

### 2.4. Co-Occurrence Network Analysis of Bacteria

**Figure 5:**
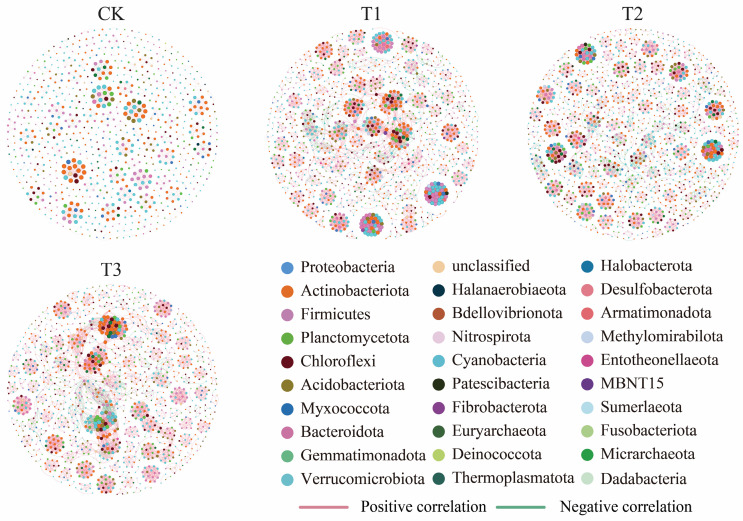
Co-occurrence network analysis of rhizosphere soil bacterial community in different CC years’ soil of the patchouli plant. CK represents zero-year cropping soil, T1 represents one year cropping soil, T2 represents two-year cropping soil, and T3 represents three-year cropping soil of patchouli. The node size represents the degree of connection, while the nodes are color-coded based on bacterial community phyla. Positive correlations are shown with red edges and negative correlations with green edges.

Co-occurrence network analysis was performed at the ASV level with a relative abundance threshold above 0.1% to investigate the interconnected traits of bacteria in the soil. The topological attributes of these networks were subsequently calculated for each treatment (Figure 5 and Table 2). Compared to the CK network, the patchouli CC networks (T1, T2, and T3) exhibited significantly higher values for nodes, edges, positive and negative correlations, average degree, average path length, and network diameter. However, the network density remained constant while the clustering coefficient decreased over the years. Positive correlations increased markedly across all treatments, with the most significant increase observed in T1 (302.40%), followed by T3 (251.35%) and T2 (223.83%). Negative correlations showed even higher relative increases, with T2 exhibiting the highest increase (228.57%), indicating an increase in antagonistic interactions within CC soils, followed by T1 (169.71%) and T3 (149.14%).

The clustering coefficient, which measured the tendency of nodes in a network to form tightly interconnected clusters, decreased across all treatments. The most significant decline was observed in T3 (17.00%), followed by T1 (8.00%) and T2 (4.00%), indicating a reduction in tightly interconnected microbial groups under CC conditions. These results showed that the CC of patchouli results in more complex microbial networks, characterized by increased interactions, path lengths, and overall connectivity, albeit with less tightly clustered microbial communities than the CK.

### 2.5. Correlations Between Soil Properties, Enzyme Activities, and Bacterial Communities

**Figure 6:**
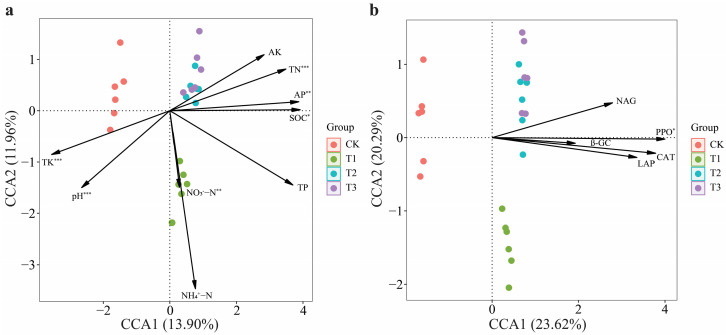
Canonical correlation analysis (CCA) of soil chemical properties, enzyme activities, and bacterial ASVs. The relationship between bacterial ASVs and soil chemical properties in the continuous cropping soil of patchouli (**a**). The relationship between bacterial ASVs and soil enzyme activities in the CC soil of patchouli (**b**). The significant differences between soil properties and bacterial ASVs are represented by * *p* < 0.05, ** *p* < 0.005, and *** *p* < 0.001.

Canonical correlation analysis (CCA) showed that various soil parameters, such as pH, SOC, NH_4_^+^–N, NO_3_^−^–N, AP, AK, TN, TP, and TK, had different effects on soil bacterial communities. Similarly, the CCA indicated that enzyme activities, including β-GC, PPO, LAP, CAT, and NAG, also influenced these bacterial communities. Among soil chemical properties, all parameters except AK, TP, and NH_4_^+^–N significantly influenced the structure of the soil bacterial communities (Figure 6a and Table S4). pH, TK, and TN had more significant effects on soil bacterial communities (*p* < 0.001 ***). Among soil enzyme activities, only PPO significantly influenced (*p* < 0.01 *) the structure of the soil bacterial communities (Figure 6b and Table S5).

**Figure 7:**
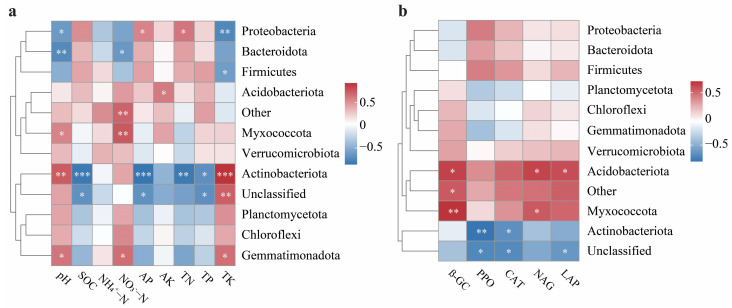
The heatmap depicts the depth of correlations between bacterial communities and soil properties at the phylum level. The heatmap shows the strength of the correlation between bacterial communities and soil chemical properties (**a**). The heatmap shows the strength of the correlation between bacterial communities and soil enzyme activities (**b**). The color gradient represents the correlation coefficients (r). The red color indicates positive correlations, and the blue indicates negative correlations. The significance differences are represented by * *p* < 0.05, ** *p* < 0.005, and *** *p* < 0.001.

The correlation heatmap analyzed the relationship between soil properties, enzyme activities, and bacterial phyla. Most bacterial phyla exhibited strong associations with soil characteristics and enzyme activities, with responses varying among the different phyla. For instance, Proteobacteria showed a negative correlation with TK and pH but a positive correlation with TN and AP (Figure 7a). Similarly, Actinobacteriota negatively correlated with TN, AP, and SOC, and positively correlated with TK and pH. Gemmatimonadota and Myxococcota exhibited positive correlations with NO_3_^−^–N and pH, while Bacteroidota showed negative correlations with these parameters. In contrast, Chloroflexi and Verrucomicrobiota demonstrated no significant correlations with soil chemical properties. Additionally, soil enzyme activities were significantly associated with various bacterial phyla (Figure 7b). Actinobacteriota showed negative correlations with PPO and CAT, whereas Myxococcota and Acidobacteriota showed positive correlations with NAG and ß-GC. Other phyla, such as Proteobacteria, Bacteroidota, Firmicutes, Planctomycetota, Chloroflexi, Gemmatimonadota, and Verrucomicrobiota, displayed no significant correlations with soil enzyme activities. Most bacterial genera demonstrated strong associations with soil properties and enzyme activities, with their responses varying across different genera. Some genera, like bacillus, positively correlated with TP, TN, AP, SOC, and PPO activities, while Longispora was negatively correlated with TP, TN, AK, AP, SOC, LAP, NAG, CAT, and PPO (Figure S2).

## 3. Discussion

### 3.1. Impact of CC on Soil Properties and the Health of Patchouli Plants

The CC alters soil properties, such as soil nutrients, organic matter, pH, and enzyme activities, which are critical for understanding its effects on soil bacterial diversity in patchouli cultivation. These factors interact and collectively influence soil properties and biodiversity [17,28,29,30]. Soil aggregates are soil’s most fundamental structural units, and their stability is a critical physical indicator for evaluating soil structure and health [31,32,33]. The soil’s physiochemical properties are correlated with the soil structure [17]. Nevertheless, they are susceptible to the impact of the cultivation system and external environmental factors, such as the intrinsic soil properties [34]. The primary physical and chemical indices that reflect soil health and fertility are soil organic matter, nutrient content, and pH, which are the primary research objects for the obstacles of CC. In this study, patchouli’s CC negatively affected soil pH, leading to gradual soil acidification. This finding aligns with observations from other studies on *Codonopsis tangshan* [35], *Polygonatum multiflorum* [36], *Cannabis sativa* [37], and *Piper nigrum* [38]. In our results on patchouli, it was found that different CC increased the contents of SOC, AP, and TN, which is aligned with the research on *Arachis hypogaea, Sesamum indicum,* and *Cucumis melo* [39,40,41]. Similarly, different CC years reduced AK, which aligns with *S. indicum* research [39]. In addition, the variation in pH in patchouli showed that long-term CC negatively affects soil’s chemical properties. The CC significantly influences the composition and activity of soil microbial communities. Research suggests that the abundance of certain beneficial microorganisms in the soil may increase with prolonged CC [42]. These microorganisms are crucial in nutrient mineralization and transformation, enhancing soil nutrient availability. For example, some microorganisms effectively decompose organic matter, releasing N and P, which can accumulate in the soil over the years [43]. Additionally, studies showed that soil acidification can influence nutrient availability by decreasing soil pH, which can enhance the solubility of certain elements, such as P, thereby increasing their effectiveness in the soil [43,44]. However, K availability is often influenced by factors such as plant uptake or salinization, and as a result, its concentration may not increase as significantly as that of other nutrients. In summary, as the duration of CC increases, most soil nutrients, except K, tend to show an upward trend. This phenomenon is primarily driven by a combination of factors, including enhanced microbial activity and changes in soil pH.

### 3.2. Response of Key Bacterial Phyla to CC in Patchouli Soil Ecosystem

Soil ecosystems host diverse bacterial communities that drive essential processes, including nutrient cycling, organic matter decomposition, and plant growth promotion [45]. Proteobacteria, one of the most abundant phyla, plays a key role in soil ecosystems with its broad metabolic abilities, including nitrogen fixation and pollutant degradation [46]. Its abundance varies with environmental conditions, agricultural practices, and soil management strategies, highlighting its adaptability and role in soil fertility and productivity. Analysis of soil microbial communities revealed significant shifts across treatments in patchouli soils, with dominant phyla like Proteobacteria, Firmicutes, Actinobacteriota, Planctomycetota, and unclassified bacteria showing relative abundances above 5% (Figure 3a). The most important bacterial phyla in CK identified in this study were Proteobacteria and Acidobacteria, consistent with findings from prior research [47]. Proteobacteria, often associated with nutrient cycling and plant growth promotion, are part of the eutrophic phyla that thrive in environments with robust nutrient availability [48]. However, a notable decline in Proteobacteria abundance was observed in treatments T1 (17.26%) and T2 (14.54%), potentially indicating that certain management practices negatively impact this beneficial group. Interestingly, a slight recovery in Proteobacteria was noted in T3 (4.87%), suggesting some resilience under specific conditions. Acidobacteria, classified as an oligotrophic group, are typically enriched in T2 (83.11%) in nutrient-deficient soils [49]. The acts of these two phyla to CC properties align with their nourishing classifications, with Proteobacteria increasing in abundance in nutrient-rich environments and Acidobacteria thriving in low-fertility conditions. Proteobacteria, among the fastest metabolizing bacteria, are vital in retaining soil environmental firmness by supplying N to the soil [50].

### 3.3. Impact of CC on Soil Bacterial Dynamics and Functional Groups

Firmicutes, known for their role in organic matter decomposition, demonstrated consistent increases across all treatments, with the most substantial rise in T1 (14.85%) [51,52]. This suggests that CC may favor the growth of Firmicutes, which are adept at thriving in nutrient-rich environments. Similarly, Actinobacteriota, which play a key role in decomposing soil organic matter and producing antibiotics to combat plant pathogens [53], initially showed high levels but experienced declines in all treatments, particularly in T3 (35.06%), raising concerns about the long-term stability of soil microbial communities under varying agricultural practices [54]. The abundance of these organisms exhibited a positive association with soil pH, contrary to the previously documented negative link between soil Actinobacteria abundance and soil pH [55]. Other notable changes were observed in Acidobacteriota and Chloroflexi, which exhibited marked increases across treatments, especially in T2 (83.11% and 58.47%, respectively). These phyla are important for organic matter decomposition and nutrient cycling, indicating that T2 may have created favorable conditions for these microbes [56]. Bacteroidota also increased significantly in T3 (50.86%), further suggesting enhanced microbial diversity and activity. Additionally, Myxococcota, important decomposers within soil ecosystems, showed significant increases in most treatments, particularly T2 (34.93%) [57]. Among these groups, Proteobacteria are generally considered the most versatile and dominant degraders due to their metabolic diversity and ability to utilize a wide range of organic compounds. They are particularly efficient in nutrient-rich environments and play a key role in rapidly decomposing organic matter [50]. Firmicutes are also key degraders, particularly in anaerobic or low-oxygen environments, where they play a significant role in breaking down complex polysaccharides and cellulose. Actinobacteriota, on the other hand, are highly effective in degrading recalcitrant organic compounds, such as lignin and chitin, and are especially active during the later stages of organic matter decomposition [53,55]. Meanwhile, Verrucomicrobiota and Gemmatimonadota displayed more moderate changes, with slight increases in T1 and T2 but declines in T3, reflecting their sensitivity to specific conditions. According to reports, Gemmatimonadota is favorably correlated with soil moisture content and is significantly impacted by the chemical characteristics of the soil [50]. However, it is more effective under conditions of higher pH, whereas acidic soil may hinder its functionality. Similar results have been observed in *Coffea arabica*, *Solanum tuberosum*, and *R. glutinosa* [58,59,60]. These findings underscored the complex interactions between soil management practices and microbial community dynamics. Understanding these relationships is essential for optimizing agricultural practices to enhance soil health and promote sustainable crop production. The mitigation methods for CC soil illnesses remain notorious, as no absolute soil controlling strategy can balance all factors, leading to repeated imbalances in the rhizosphere microecosystem. The vibrant changes in root exudate components clearly affect the assembly and behavior of rhizosphere microorganisms [61], highlighting the need for further research on the complex interactions between root exudation, soil, and microbes. Developing specialized microbial fertilizers for crops like patchouli is crucial for creating a healthy soil environment. These findings underscore the complex interactions between soil management practices and microbial community dynamics. Understanding these relationships is essential for optimizing agricultural practices to enhance soil health and promote sustainable crop production.

## 4. Materials and Methods

### 4.1. Experimental Site and Design

The experiment was conducted at the School of Breeding and Multiplication, Hainan University, Sanya, China (18°2′5″ N, 109°09′14″ E), using the “Nanxiang” cultivar obtained from the germplasm resource garden in Haikou (20°3′35″ N, 110°19′8″ E). Vigorous, pest-free *P. cablin* branches were propagated via cuttings in a sterilized sandbed for 60 days. The uniform seedlings were transplanted into 72 pots, each with a dimension of L22 cm × W20 cm × H15 cm. The pots were manually filled using a hand tool (shovel) with 5 kg of a soil mixture comprising organic fertilizer (sheep manure) and laterite soil in a 1:6 ratio. The 4 different CC year soils were used: zero-year (CK, control; soil where patchouli plants had not been grown previously), one-year CC soil of patchouli (T1), two-year CC soil of patchouli (T2), and three-year CC soil of patchouli (T3). Each treatment consisted of 18 pots, arranged in 3 replicates, with each replicate containing 6 pots per treatment in a randomized complete block design. These 4 different CC patchouli seedling treatments were grown under greenhouse conditions for 3 months, exposed to natural sunlight, and maintained at temperatures ranging from 20 °C to 25 °C. The 3-month duration was selected based on this group’s previous research, which identified the rapid growth stage as being most affected by CC [62].

### 4.2. Soil Samples Collection

Soil samples from the patchouli plant were collected according to the methodology described by Ahmed et al. to evaluate soil properties, enzyme activities, and rhizosphere bacterial diversity [63]. Tiny soil particles adhering to the root surface were carefully brushed off using a soft nylon brush with fine bristles (dimensions: L20.5 × W9.5 × H1.5 cm) [63]. This brush is ideal for collecting rhizosphere soil samples, as it removes soil without damaging the roots or altering the microbial composition. Six rhizosphere soils (collected from six plants per treatment) were combined into a single sample per replicate, with three replicates per treatment. Samples were preserved at −80 °C and room temperature for subsequent laboratory analyses. Additionally, a portion of the fresh soil was preserved at 4 °C for 24 h for soil enzyme activity analysis.

### 4.3. Soil Chemical Properties and Enzyme Activities

To examine the soil’s chemical properties, the soil pH was assessed utilizing a pH electrode (Mettler Toledo, United States) in a soil–water ratio (1:2.5 *w*/*v*) after 30 min of mixing. Soil organic carbon (SOC; g kg^−1^) was examined with the help of the K_2_Cr_2_O_7_.H_2_SO_4_ method established by Nelson and Sommers [64]. Soil ammonium nitrogen (NH_4_^+^-N; mg kg^−1^) and nitrate nitrogen (NO_3_^−^-N; mg kg^−1^) were extracted with 2.00 M KCl and assayed using a continuous flow analytical system (SAN, SKALAR, Breda, The Netherlands) [65,66]. Soil-available potassium (AK; mg kg^−1^) and available phosphorus (AP; mg kg^−1^) extracted with 1.00 M CH_3_COONH_4_ and 0.50 M NaHHCO_3_, respectively, were quantified by inductively coupled plasma-atomic emission spectrometry (ICPS-7500, Shimadzu, Kyoto, Japan) [67]. The soil total potassium (TK; g kg^−1^), total phosphorus (TP; g kg^−1^), and total nitrogen (TN; g kg^−1^) were determined by the elemental tester (VarioEL III, Elementar, Hanau, Germany) and H_2_SO_4_.HClO_4_ digestion method [68]. Similarly, five soil enzyme activities, ß-Glucosidase (ß-GC; nmol h^−1^ g^−1^), polyphenol oxidase (PPO; nmol h^−1^ g^−1^), catalase (CAT; µmol h^−1^ g^−1^), N-acetyl-β-D-glucosaminidase (NAG; nmol h^−1^ g^−1^), and leucine aminopeptidase (LAP; nmol h^−1^ g^−1^), were analyzed using assay kits (Suzhou Geruisi, Biotechnology, Co., Ltd., Suzhou, China) following the manufacturer protocols. The absorbance readings were measured using a spectrophotometer (Spectrum, SP-756P, California, USA) at specific wavelengths: NAG (Cat. no. G0321F), LAP (Cat. no. G0329F), and ß-GC (Cat. no. G0312F) at 405 nm, PPO (Cat. no. G0311F) at 475 nm, and CAT (Cat. no. G0303F) at 510 nm.

### 4.4. Soil DNA Extraction, PCR Amplification, and 16S Sequencing

The soil DNA from the frozen rhizosphere soil samples was obtained via the SPINeasy™ DNA Pro Kit for Soil (Cat. No.: 116546050, MP Biomedicals, Irvine, CA, USA), according to the manufacturer’s information [69]. The DNA quality and concentration were measured using a NanoDrop^®^ ND-2000 spectrophotometer (Thermo Scientific, Waltham, MA, USA). The V3-V4 region of the bacterial 16S rRNA gene was amplified for sequencing using the forward primer 341F (5′-CCTACGGGNGGCWGCAG-3′) and the reverse primer 806R (5′-GGACTACHVGGGTATCTAAT-3′) [70,71]. Sequencing libraries were prepared using the Illumina DNA Prep Kit (Illumina, CA, USA) according to the manufacturer’s protocols, and their quality was evaluated with the ABI StepOnePlus RT-PCR System (Life Technologies, Foster City, CA, USA). Finally, 2 × 250 bp paired-end reads were spawned on the Novaseq 6000 platform at BGI Genomics Co., Ltd., Shenzhen, China.

### 4.5. Bioinformatics Analysis

FASTP (v0.18.0) filtered raw reads were based on defined criteria, ensuring high-quality clean reads while eliminating adapter sequences and squat-quality data that could alter attachment and finding [72]. FLASH (v1.2.11) was then used to merge paired-end clean reads, requiring at least a 10 bp overlap with a maximum mismatch rate of 2% [73]. To acquire superior-quality clean tags, erroneous sequences from the raw tags were filtered in a controlled environment [74]. The clean tags were then clustered into ASVs (amplicon sequence variants) using the UPARSE pipeline (v9.2.64) [75]. Chimeric tags were removed using the UCHIME algorithm to obtain effective tags for further analysis [76]. The tag sequence with the highest abundance was selected as the representative sequence for each cluster. Principal coordinate analysis (PCoA) based on Bray–Curtis distance was conducted using the “vegan” package in R (v4.4.1). Boxplot analysis utilized the “ggpubr” and “Hmisc” packages, with visualization from “corrplot.” Correlation network analysis and stacked diagrams were generated using microbial abundance data from the cloud platform (www.omicsshare.com). A volcano plot was created via differential analysis with the “Deseq2” package and visualized using “ggplot2.” Venn diagrams were drawn using tools from the cloud platform (www.cnsknowall.com). Canonical correlation analysis (CCA) was performed with the “GGally” and “CCA” packages, also visualized with “ggplot2.” The co-occurrence network of patchouli rhizosphere microorganisms was constructed using “Hmisc” and visualized in Gephi version 0.9.2, with nodes and edges representing specific species. The statistical significance of soil properties, α-bacterial diversity, and relative abundance across taxonomic ranks was assessed using analysis of variance (ANOVA), with a joint relation using Tukey’s HSD test.

## 5. Conclusions

In summary, patchouli’s CC significantly impacted the soil properties and enzyme activities. Soil pH slightly decreased over the years of CC, indicating a trend toward acidification. Other indicators like SOC, AK, AP, and TP significantly increased. Similarly, enzyme activities ß-GC, PPO, CAT, LAP, and NAG remarkably increased until the second year of CC, followed by a decline in the third year. The diversity of rhizosphere bacteria decreased substantially despite increasing their relative abundance under CC conditions. CC altered bacterial community structure and composition, reducing the relative abundance of Proteobacteria, Firmicutes, Actinobacteria, and Planctomycetota over time. The loss of diversity in soil bacterial communities might be the key cause of the lower production, quality, and soil-borne illnesses in *P. cablin*. The findings provide important evidence on the variations in soil bacterial communities in the CC of *P. cablin*. Applying microbial techniques, such as investigating soil amendments and bacteriological stimulants, should be prioritized during patchouli production to mitigate ongoing cropping soil obstacles.

## Figures and Tables

**Table 1:** Impact of patchouli CC on soil chemical properties.

Treatment	CK	T1	T2	T3
pH	8.21 ± 0.04 a	8.13 ± 0.06 a	8.08 ± 0.05 a	7.85 ± 0.10 b
SOC (g kg^−1^)	8.70 ± 0.13 a	31.70 ± 0.80 b	35.85 ± 1.21 c	40.90 ± 1.73 d
NH_4_^+^–N (mg kg^−1^)	8.77 ± 0.43 a	10.95 ± 0.38 b	8.88 ± 0.43 b	8.67 ± 0.49 b
NO_3_^−^–N (mg kg^−1^)	13.76 ± 0.49 a	15.01 ± 1.18 a	14.89 ± 0.78 ab	11.27 ± 0.93 b
AK (mg kg^−1^)	119.56 ± 0.85 a	171.96 ± 1.18 b	371.86 ± 2.47 c	221.64 ± 1.36 d
AP (mg kg^−1^)	29.76 ± 1.74 a	117.10 ± 1.00 b	130.15 ± 0.51 c	161.60 ± 4.55 d
TK (g kg^−1^)	0.77 ± 0.02 a	0.71 ± 0.03 b	0.69 ± 0.02 b	0.65 ± 0.02 c
TN (g kg^−1^)	0.24 ± 0.03 a	0.83 ± 0.08 b	0.95 ± 0.06 b	1.45 ± 0.02 c
TP (g kg^−1^)	0.35 ± 0.01 a	1.85 ± 0.03 b	1.62 ± 0.02 b	1.53 ± 0.11 c

Note: Mean value ± S.D. pH; soil pH, SOC; soil organic carbon, NH_4_^+^–N; ammonium-nitrogen, NO_3_^−^–N; nitrate-nitrogen, AK; available potassium, AP; available phosphorous, TK; total potassium, TN; total nitrogen, and TP for total phosphorous. CK; zero-year cropping soil (Control), T1; one-year cropping soil, T2; two-year cropping soil, and T3; three-year cropping soil. Significant variations among the four “treatments” are signaled by unique small letter alphabets (*p* < 0.05) following Tukey’s HSD tests.

**Table 2:** Co-occurrence network analysis features of the different CC soil of patchouli.

Network Indicator	CK	T1	T2	T3
Node	816	1271	1323	1204
Edge	1803	7023	5847	6156
Positive correlation	1628	6551	5272	5720
Negative correlation	175	472	575	436
Average degree	4.42	11.05	8.84	10.23
Average path length	1.00	8.60	6.23	10.24
Network diameter	1.00	33.50	26.61	33.51
Network density	0.01	0.01	0.01	0.01
Clustering coefficient	1.00	0.92	0.96	0.83

## Data Availability

The data will be made available on request.

## Supplementary Materials

The following supporting information can be downloaded at: https://www.mdpi.com/article/10.3390/plants13243481/s1. Figure S1. Impact of patchouli CC on the diversity of soil bacteria. The Simpson (a), Chao (b), Pielou (c), and Pd (d) index bacterial diversity in the rhizosphere soil of the patchouli plant. The significance differences represented by ns: non-significant, * *p* < 0.05, ** *p* < 0.005, and *** *p* < 0.001. Figure S2. The heatmap depicts the depth of correlations between bacterial communities and soil properties at the genus level. The heatmap showed the strength of the correlation between bacterial communities and soil chemical properties (a). The heatmap showed the strength of the correlation between bacterial communities and soil enzyme activities (b). The color gradient represents the correlation coefficients (r). The red color indicates positive correlations, and the blue indicates negative correlations. The significance differences are represented by * *p* < 0.05, ** *p* < 0.005, and *** *p* < 0.001. Table S1. All ASVs obtained by 16S rRNA sequences of all different CC years soil samples of patchouli. Table S2. The predominant bacterial phyla in all different CC year soil samples of patchouli. Table S3. The predominant bacterial genera in all different CC years soil samples of patchouli. Table S4. The bacterial ASVs correlation between soil chemical properties in different CC soil of patchouli. Table S5. The bacterial ASVs correlation between soil enzyme activities in different CC soil of patchouli.

## References

[1] Mo M., Lin W., Haq M.Z.U., Wang Y., Yang E., Yu J., Xia P. (2024). Exogenous glutathione (GSH/GSSG) promoted the synthesis of patchoulol and pogostone, main active components of *Pogostemon cablin* (Patchouli). Ind. Crops Prod..

[2] Liu X., Zeeshan Ul Haq M., Yu J., Liu Y., Yang H., Cui H., Yang D., Wu Y. (2023). Identification of the CDPK gene family in patchouli and functional analysis in response to continuous cropping stress. Front. Plant Sci..

[3] Yan W., Liu X., Cao S., Yu J., Zhang J., Yao G., Yang H., Yang D., Wu Y. (2023). Molecular basis of *Pogostemon cablin* responding to continuous cropping obstacles revealed by integrated transcriptomic, miRNA and metabolomic analyses. Ind. Crops Prod..

[4] Zeeshan Ul Haq M., Yu J., Yao G., Yang H., Iqbal H.A., Tahir H., Cui H., Liu Y., Wu Y. (2023). A systematic review on the continuous cropping obstacles and control strategies in medicinal plants. Int. J. Mol. Sci..

[5] Zeng J., Liu J., Lu C., Ou X., Luo K., Li C., He M., Zhang H., Yan H. (2020). Intercropping with turmeric or ginger reduce the continuous cropping obstacles that affect *Pogostemon cablin* (patchouli). Front. Microbiol..

[6] Li J., Cheng X., Chu G., Hu B., Tao R. (2023). Continuous cropping of cut chrysanthemum reduces rhizospheric soil bacterial community diversity and co-occurrence network complexity. Appl. Soil Ecol..

[7] Xiao Z., Lu C., Wu Z., Li X., Ding K., Zhu Z., Han R., Zhao J., Ge T., Li G. (2024). Continuous cropping disorders of eggplants (Solanum melongena L.) and tomatoes (*Solanum lycopersicum* L.) in suburban agriculture: Microbial structure and assembly processes. Sci. Total Environ..

[8] Xiong J.-X., Du L.-S., Li N.-N., Wu X.-T., Xiang Y., Li S., Zou L., Liu D., Huang D., Xie Z.F. (2024). *Pigmentiphaga kullae* CHJ604 improved the growth of tobacco by degrading allelochemicals and xenobiotics in continuous cropping obstacles. J. Hazard. Mater..

[9] Yan W., Cao S., Wu Y., Ye Z., Zhang C., Yao G., Yu J., Yang D., Zhang J. (2022). Integrated analysis of physiological, mRNA sequencing, and miRNA sequencing data reveals a specific mechanism for the response to continuous cropping obstacles in *Pogostemon cablin* roots. Front. Plant Sci..

[10] Li J., Chen X., Li S., Zuo Z., Zhan R., He R. (2020). Variations of rhizospheric soil microbial communities in response to continuous *Andrographis paniculata* cropping practices. Bot. Stud..

[11] Xu Y., Wu Y.-G., Chen Y., Zhang J.-F., Song X.-Q., Zhu G.-P., Hu X.-W. (2015). Autotoxicity in *Pogostemon cablin* and their allelochemicals. Rev. Bras. De Farmacogn..

[12] Wang L., Lu P., Feng S., Hamel C., Sun D., Siddique K.H., Gan G.Y. (2024). Strategies to improve soil health by optimizing the plant–soil–microbe–anthropogenic activity nexus. Agric. Ecosyst. Environ..

[13] Faizan M., Singh A., Eren A., Sultan H., Sharma M., Djalovic I., Trivan G. (2024). Small molecule, big impacts: Nano-nutrients for sustainable agriculture and food security. J. Plant Physiol..

[14] Liao J., Xia P. (2024). Continuous cropping obstacles of medicinal plants: Focus on the plant-soil-microbe interaction system in the rhizosphere. Sci. Hortic..

[15] Philippot L., Chenu C., Kappler A., Rillig M.C., Fierer N. (2024). The interplay between microbial communities and soil properties. Nat. Rev. Microbiol..

[16] Liu S., Wang Z., Niu J., Dang K., Zhang S., Wang S., Wang Z. (2021). Changes in physicochemical properties, enzymatic activities, and the microbial community of soil significantly influence the continuous cropping of *Panax quinquefolius* L. (American ginseng). Plant Soil.

[17] Pervaiz Z.H., Iqbal J., Zhang Q., Chen D., Wei H., Saleem M. (2020). Continuous cropping alters multiple biotic and abiotic indicators of soil health. Soil Syst..

[18] Song R., Zhu W.Z., Li H., Wang H. (2024). Impact of wine-grape continuous cropping on soil enzyme activity and the composition and function of the soil microbial community in arid areas. Front. Microbiol..

[19] Wang F., Zhang X., Wei M., Wang Y., Liang Z., Xia P. (2022). Appropriate crop rotation alleviates continuous cropping barriers by changing rhizosphere microorganisms in *Panax notoginseng*. Rhizosphere.

[20] Wang R., Xiao Y., Lv F., Hu L., Wei L., Yuan Z., Lin H. (2018). Bacterial community structure and functional potential of rhizosphere soils as influenced by nitrogen addition and bacterial wilt disease under continuous sesame cropping. Appl. Soil Ecol..

[21] Zhang S., Jiang Q., Liu X., Liu L., Ding W. (2020). Plant growth promoting rhizobacteria alleviate aluminum toxicity and ginger bacterial wilt in acidic continuous cropping soil. Front. Microbiol..

[22] Wu D., Wang W., Yao Y., Li H., Wang Q., Niu B. (2023). Microbial interactions within beneficial consortia promote soil health. Sci. Total Environ..

[23] Daunoras J., Kačergius A., Gudiukaitė R. (2024). Role of soil microbiota enzymes in soil health and activity changes depending on climate change and the type of soil ecosystem. Biology.

[24] Zuccarini P., Sardans J., Asensio L., Peñuelas J. (2023). Altered activities of extracellular soil enzymes by the interacting global environmental changes. Glob. Change Biol..

[25] Wang L., Hamel C., Lu P., Wang J., Sun D., Wang Y., Lee S.-J., Gan G.Y. (2023). Using enzyme activities as an indicator of soil fertility in grassland-an academic dilemma. Front. Plant Sci..

[26] Wang J.-T., Zhang Y.-B., Xiao Q., Zhang L.-M. (2022). Archaea is more important than bacteria in driving soil stoichiometry in phosphorus deficient habitats. Sci. Total Environ..

[27] Zhang Y., Guo R., Li S., Chen Y., Li Z., He P., Huang X., Huang K. (2021). Effects of continuous cropping on soil, senescence, and yield of Tartary buckwheat. Agron. J..

[28] Wang Y., Xu X., Liu T., Wang H., Yang Y., Chen X., Zhu S. (2020). Analysis of bacterial and fungal communities in continuous-cropping ramie (*Boehmeria nivea* L. Gaud) fields in different areas in China. Sci. Rep..

[29] Wang W., Wang N., Dang K., Dai W., Guan L., Wang B., Gao J., Cui Z., Dong Y., Wang H. (2020). Long-term nitrogen application decreases the abundance and copy number of predatory myxobacteria and alters the myxobacterial community structure in the soil. Sci. Total Environ..

[30] Yang J.-i., Ruegger P.M., McKenry M.V., Becker J.O., Borneman J. (2012). Correlations between root-associated microorganisms and peach replant disease symptoms in a California soil. PLoS ONE.

[31] Abiven S., Menasseri S., Chenu C. (2009). The effects of organic inputs over time on soil aggregate stability—A literature analysis. Soil Biol. Biochem..

[32] Almajmaie A., Hardie M., Acuna T., Birch C. (2017). Evaluation of methods for determining soil aggregate stability. Soil Tillage Res..

[33] Wang Y., Zou L., Lou C., Geng X., Zhang S., Chen X., Zhang Y., Huang D., Liang A. (2024). No-tillage with straw retention influenced maize root growth morphology by changing soil physical properties and aggregate structure in Northeast China: A ten-year field experiment. Geoderma Reg..

[34] Dong X., Wang J., Tian L., Lou B., Zhang X., Liu T., Liu X., Sun H. (2023). Review of relationships between soil aggregates, microorganisms and soil organic matter in salt-affected soil. Chin. J. Eco-Agric..

[35] Zhang M., He Y., Zhou W., Ai L., Liu H., Chen L., Xie Y. (2021). Effects of continuous cropping of *Codonopsis tangshen* on rhizospheric soil bacterial community as determined by pyrosequencing. Diversity.

[36] Alami M.M., Pang Q., Gong Z., Yang T., Tu D., Zhen O., Yu W., Alami M.J., Wang X. (2021). Continuous cropping changes the composition and diversity of bacterial communities: A meta-analysis in nine different fields with different plant cultivation. Agriculture.

[37] Guo L., Chen X., Li Z., Wang M., Che Y., Zhang L., Jiang Z., Jie S. (2022). Effects of continuous cropping on bacterial community and diversity in rhizosphere soil of industrial hemp: A five-year experiment. Diversity.

[38] Xiong W., Li Z., Liu H., Xue C., Zhang R., Wu H., Li R., Shen Q. (2015). The effect of long-term continuous cropping of black pepper on soil bacterial communities as determined by 454 pyrosequencing. PLoS ONE.

[39] Wacal C., Ogata N., Basalirwa D., Sasagawa D., Ishigaki T., Handa T., Kato M., Tenywa M.M., Masunaga T., Yamamoto S. (2019). Imbalanced soil chemical properties and mineral nutrition in relation to growth and yield decline of sesame on different continuously cropped upland fields converted paddy. Agronomy.

[40] Li H., Li C., Song X., Liu Y., Gao Q., Zheng R., Li J., Zhang P., Liu X. (2022). Impacts of continuous and rotational cropping practices on soil chemical properties and microbial communities during peanut cultivation. Sci. Rep..

[41] Wang J., Li M., Zhou Q., Zhang T. (2023). Effects of continuous cropping Jiashi muskmelon on rhizosphere microbial community. Front. Microbiol..

[42] Li Y., Shi C., Wei D., Gu X., Wang Y., Sun L., Cai S., Hu Y., Jin L., Wang W. (2022). Soybean continuous cropping affects yield by changing soil chemical properties and microbial community richness. Front. Microbiol..

[43] Wang K., Lu Q., Dou Z., Chi Z., Cui D., Ma J., Wang G., Kuang J., Wang N., Zuo Y. (2024). A review of research progress on continuous cropping obstacles. Front. Agric. Sci. Eng..

[44] Wu H., Lin W. (2020). A commentary and development perspective on the consecutive monoculture problems of medicinal plants. Chin. J. Eco-Agric..

[45] Yadav A.N., Kour D., Kaur T., Devi R., Yadav A., Dikilitas M., Abdel-Azeem A.M., Ahluwalia A.S., Saxena A.K. (2021). Biodiversity, and biotechnological contribution of beneficial soil microbiomes for nutrient cycling, plant growth improvement and nutrient uptake. Biocatal. Agric. Biotechnol..

[46] Gao T., Wang Y., Lai J., Wang F., Yao G., Bao S., Liu J., Wan X., Chen C., Zhang Y. (2024). Effects of nitrile compounds on the structure and function of soil microbial communities as revealed by metagenomes. Environ. Res..

[47] Li Y., Li Z., Li Z., Jiang Y., Weng B., Lin W. (2016). Variations of rhizosphere bacterial communities in tea (*Camellia sinensis* L.) continuous cropping soil by high-throughput pyrosequencing approach. J. Appl. Microbiol..

[48] Dai Z., Su W., Chen H., Barberán A., Zhao H., Yu M., Yu L., Brookes P.C., Schadt C.W., Chang S.X. (2018). Long-term nitrogen fertilization decreases bacterial diversity and favors the growth of Actinobacteria and Proteobacteria in agro-ecosystems across the globe. Glob. Change Biol..

[49] Ai C., Zhang S., Zhang X., Guo D., Zhou W., Huang S. (2018). Distinct responses of soil bacterial and fungal communities to changes in fertilization regime and crop rotation. Geoderma.

[50] Ren H., Huang B., Fernández-García V., Miesel J., Yan L., Lv C. (2020). Biochar and rhizobacteria amendments improve several soil properties and bacterial diversity. Microorganisms.

[51] Liu Y., Wang W., Shah S.B., Zanaroli G., Xu P., Tang H. (2020). Phenol biodegradation by Acinetobacter radioresistens APH1 and its application in soil bioremediation. Appl. Microbiol. Biotechnol..

[52] Radolfova-Krizova L., Maixnerova M., Nemec A. (2016). *Acinetobacter pragensis* sp. nov., found in soil and water ecosystems. Int. J. Syst. Evol. Microbiol..

[53] Zhang H., Wang R., Chen S., Qi G., He Z., Zhao X. (2017). Microbial taxa and functional genes shift in degraded soil with bacterial wilt. Sci. Rep..

[54] Chen M.-M., Zhu Y.-G., Su Y.-H., Chen B.-D., Fu B.-J., Marschner P. (2007). Effects of soil moisture and plant interactions on the soil microbial community structure. Eur. J. Soil Biol..

[55] el Zahar Haichar F., Santaella C., Heulin T., Achouak W. (2014). Root exudates mediated interactions belowground. Soil Biol. Biochem..

[56] Nesme J., Simonet P. (2015). The soil resistome: A critical review on antibiotic resistance origins, ecology and dissemination potential in telluric bacteria. Environ. Microbiol..

[57] Liu W., Wang N., Yao X., He D., Sun H., Ao X., Wang H., Zhang H., St. Martin S., Xie F. (2023). Continuous-cropping-tolerant soybean cultivars alleviate continuous cropping obstacles by improving structure and function of rhizosphere microorganisms. Front. Microbiol..

[58] Liu X., Zhang J., Gu T., Zhang W., Shen Q., Yin S., Qiu H. (2014). Microbial community diversities and taxa abundances in soils along a seven-year gradient of potato monoculture using high throughput pyrosequencing approach. PLoS ONE.

[59] Wu L., Chen J., Xiao Z., Zhu X., Wang J., Wu H., Wu Y., Zhang Z., Lin W. (2018). Barcoded pyrosequencing reveals a shift in the bacterial community in the rhizosphere and rhizoplane of *Rehmannia glutinosa* under consecutive monoculture. Int. J. Mol. Sci..

[60] Zhao Q., Xiong W., Xing Y., Sun Y., Lin X., Dong Y. (2018). Long-term coffee monoculture alters soil chemical properties and microbial communities. Sci. Rep..

[61] Vives-Peris V., De Ollas C., Gómez-Cadenas A., Pérez-Clemente R.M. (2020). Root exudates: From plant to rhizosphere and beyond. Plant Cell Rep..

[62] Yan W., Cao S., Liu X., Yao G., Yu J., Zhang J., Bian T., Yu W., Wu Y. (2022). Combined physiological and transcriptome analysis revealed the response mechanism of *Pogostemon cablin* roots to p-hydroxybenzoic acid. Front. Plant Sci..

[63] Ahmed W., Dai Z., Zhang J., Shakeel Q., Kamaruzzaman M., Nosheen S., Mohany M., Ahmed A., Cai S., Wang Y. (2024). *Ralstonia solanacearum* differentially modulates soil physicochemical properties and rhizospheric bacteriome of resistant and susceptible tobacco cultivars. Microbiol. Res..

[64] Nelson D.W., Sommers L.E. (1996). Total carbon, organic carbon, and organic matter. Methods Soil Anal. Part 3 Chem. Methods.

[65] Dospatliev L., Ivanova M. (2022). Inductively coupled plasma optical emission spectrometry determination of total phosphorus AND sulphur in virginia tobacco leaves. Oxid. Commun..

[66] Miranda K.M., Espey M.G., Wink D.A. (2001). A rapid, simple spectrophotometric method for simultaneous detection of nitrate and nitrite. Nitric Oxide.

[67] Dahlquist R., Knoll J. (1978). Inductively coupled plasma-atomic emission spectrometry: Analysis of biological materials and soils for major, trace, and ultra-trace elements. Appl. Spectrosc..

[68] Jones D.L., Willett V.B. (2006). Experimental evaluation of methods to quantify dissolved organic nitrogen (DON) and dissolved organic carbon (DOC) in soil. Soil Biol. Biochem..

[69] Sun Y., Teng Y., Li R., Wang X., Zhao L. (2024). Microbiome resistance mediates stimulation of reduced graphene oxide to simultaneous abatement of 2,2′,4,4′,5-pentabromodiphenyl ether and 3,4-dichloroaniline in paddy soils. J. Hazard. Mater..

[70] Guo M., Wu F., Hao G., Qi Q., Li R., Li N., Wei L., Chai T. (2017). Bacillus subtilis improves immunity and disease resistance in rabbits. Front. Immunol..

[71] Walters W., Hyde E.R., Berg-Lyons D., Ackermann G., Humphrey G., Parada A., Gilbert J.A., Jansson J.K., Caporaso J.G., Fuhrman J.A. (2016). Improved bacterial 16S rRNA gene (V4 and V4-5) and fungal internal transcribed spacer marker gene primers for microbial community surveys. mSystems.

[72] Chen S., Zhou Y., Chen Y., Gu J. (2018). fastp: An ultra-fast all-in-one FASTQ preprocessor. Bioinformatics.

[73] Magoč T., Salzberg S.L. (2011). FLASH: Fast length adjustment of short reads to improve genome assemblies. Bioinformatics.

[74] Bokulich N.A., Subramanian S., Faith J.J., Gevers D., Gordon J.I., Knight R., Mills D.A., Caporaso J.G. (2013). Quality-filtering vastly improves diversity estimates from Illumina amplicon sequencing. Nat. Methods.

[75] Edgar R.C. (2013). UPARSE: Highly accurate OTU sequences from microbial amplicon reads. Nat. Methods.

[76] Edgar R.C., Haas B.J., Clemente J.C., Quince C., Knight R. (2011). UCHIME improves sensitivity and speed of chimera detection. Bioinformatics.

