# Supplementary material for: Continuous Cropping of Patchouli Alleviate Soil Properties, Enzyme Activities, and Bacterial Community Structures
Source: Plants (Basel). 2024 Dec 12;13(24):3481. doi: 10.3390/plants13243481 (PMC11728537; doi:10.3390/plants13243481)
Supplement: Supplementary file 1 [file plants-13-03481-s001.zip › plants-3310720-supplementary.pdf]

Supplementary Figures and Tables

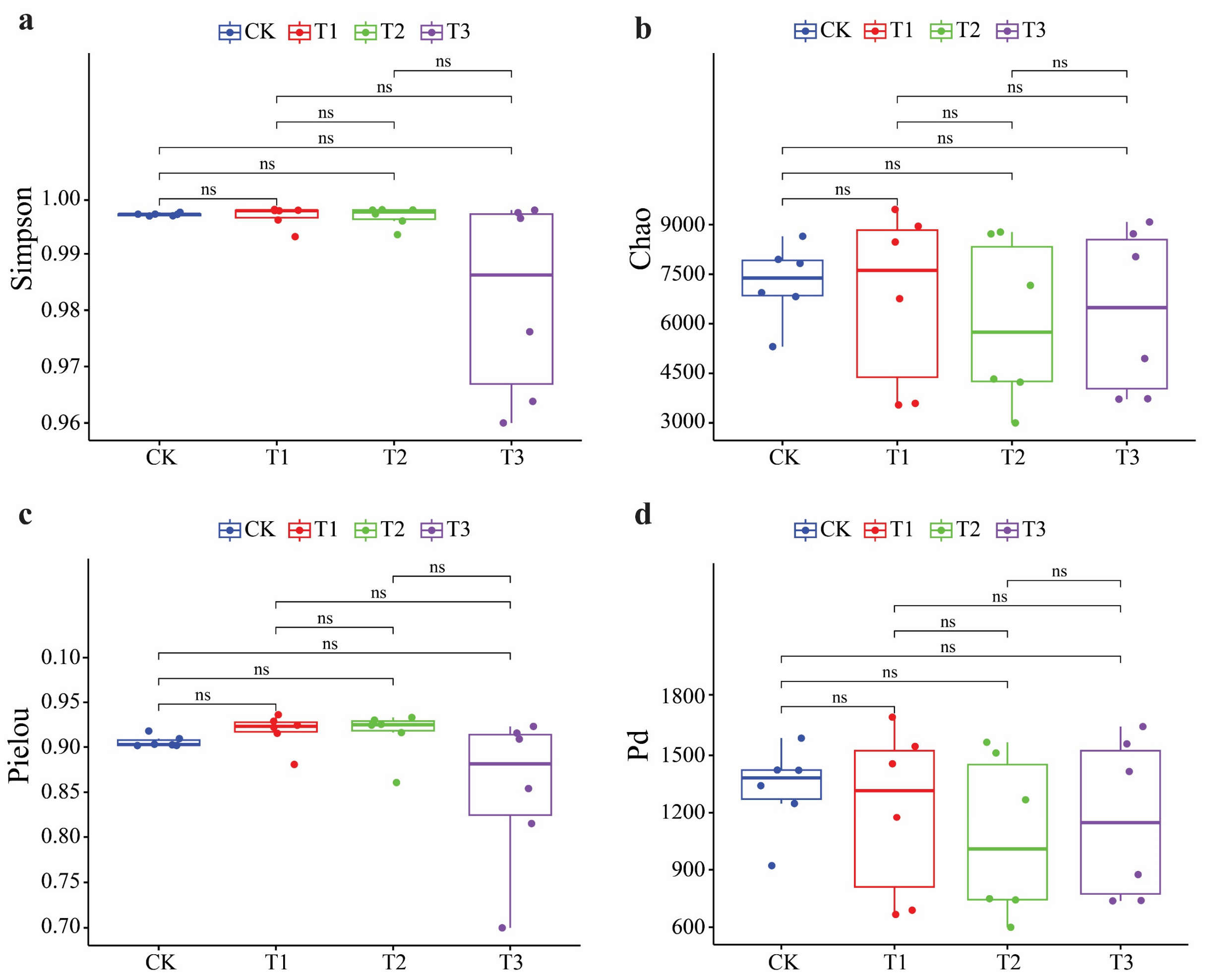

Figure S1. Impact of patchouli CC on the diversity of soil bacteria. The Simpson (a), Chao (b), Pielou (c), and Pd (d) index bacterial diversity in the rhizosphere soil of the patchouli plant. The significance differences represented by ns: non-significant, * $p < 0.05$, ** $p < 0.005$, and *** $p < 0.001$.

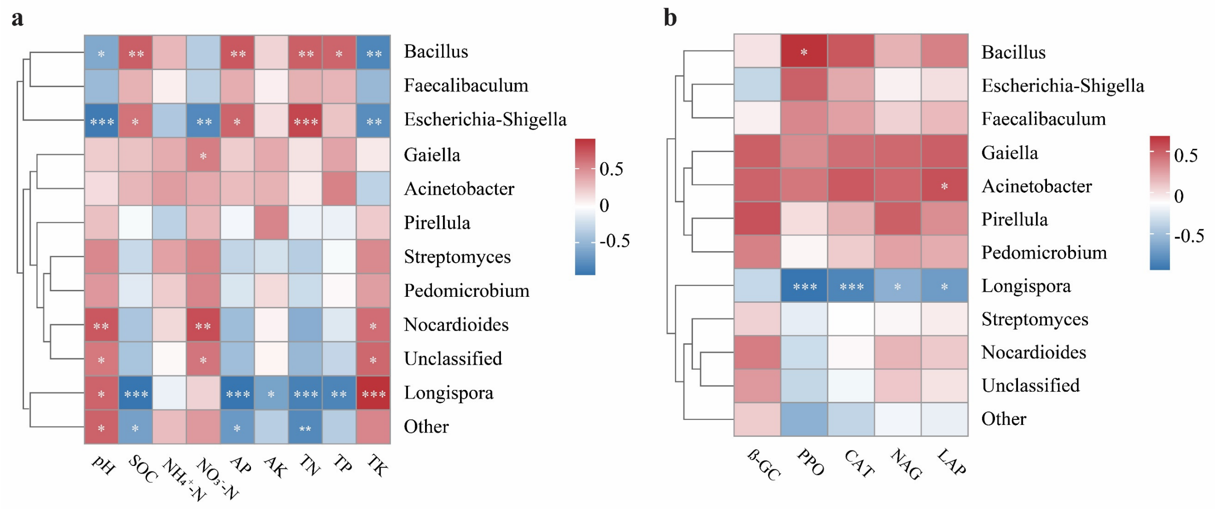

Figure S2. The heatmap depicts the depth of correlations between bacterial communities and soil properties at the genus level. The heatmap showed the strength of the correlation between bacterial communities and soil chemical properties (a). The heatmap showed the strength of the correlation between bacterial communities and soil enzyme activities (b). The color gradient represents the correlation coefficients (r). The red color indicates positive correlations, and the blue indicates negative correlations. The significance differences are represented by * $p < 0.05$, ** $p < 0.005$, and *** $p < 0.001$.

Table S1. All ASVs obtained by 16S rRNA sequences of all different CC years soil samples of patchouli.

Sample ID	Total Tags	Taxon Tags	Unclassified Tags	Singleton Tags	ASVs
CK-1	69843	69843	0	0	4682
CK-2	67654	67654	0	0	6197
CK-3	75183	75183	0	0	7322
CK-4	62248	62244	4	0	7202
CK-5	70780	70778	2	0	8023
CK-6	56395	56392	3	0	6316
T1-1	52656	52656	0	0	2916
T1-2	65611	65611	0	0	2964
T1-3	73204	73204	0	0	6133
T1-4	78066	78066	0	0	7848
T1-5	68559	68559	0	0	8328
T1-6	69747	69747	0	0	8835
T2-1	71467	71467	0	0	3606
T2-2	61666	61666	0	0	2374
T2-3	84276	84276	0	0	3703
T2-4	69152	69152	0	0	6536
T2-5	76207	76207	0	0	8151
T2-6	66183	66183	0	0	8094
T3-1	67798	67798	0	0	3093
T3-2	61323	61323	0	0	3106
T3-3	65029	65029	0	0	4323
T3-4	79310	79310	0	0	7405
T3-5	70497	70497	0	0	8097
T3-6	68977	68977	0	0	8454
**Total**	**1651831**				**143708**

Table S2. The predominant bacterial phyla in all different CC year soil samples of patchouli.

Phylum	CK	T1	T2	T3
Proteobacteria	22.38171	18.51769	19.1282	23.47077
Firmicutes	17.76436	20.40216	19.31255	19.38852
Actinobacteriota	23.22219	19.81957	17.61474	15.08055
Planctomycetota	6.440611	6.451658	6.90377	7.298754
Acidobacteriota	3.961578	6.118691	7.253949	6.574153
Chloroflexi	4.276886	5.662149	6.777483	5.376862
Bacteroidota	3.722602	3.120364	4.164401	5.615858
Myxococcota	2.828771	3.462583	3.816844	2. 79112
Pirellula	1.781845	1.925274	1.674584	1.624235
Gemmatimonadota	1.508555	1.873289	1.777534	1.367573
Other	3.921746	4.838796	4.233812	3.829655
Unclassified	8.189156	7.80778	7.342141	7.581945

Table S3. The predominant bacterial genera in all different CC years soil samples of patchouli.

Genus	CK	T1	T2	T3
Bacillus	1.765441	4.79406	5.651512	5.32424
Escherichia-Shigella	0.076137	0.347292	1.634144	9.133605
Gaiella	0.734943	1.661432	1.502103	1.365137
Longispora	2.380942	1.322002	1.005969	0.749383
Faecalibaculum	0.60385	1.705024	1.118061	1.85853
Acinetobacter	0.22934	0.770361	0.623897	0.365821
Streptomyces	0.639137	0.928421	0.595809	0.599531
Nocardioides	0.681832	0.802976	0.850332	0.593879
Pirellula	0.723336	0.617935	0.853653	0.929202
Pedomicrobium	0.566675	0.689482	0.611333	0.637478
Other	38.797111	29.396633	26.875577	24.12547
Unclassified	52.801256	56.961037	58.677609	54.317723

Table S4. The bacterial ASVs correlation between soil chemical properties in different CC soil of patchouli.

***VECTORS					
	**ASV000001**	**ASV000002**	**r^2**	**Pr (> r)**	
pH	-0.95822	0.28603	0.6585	0.001	***
SOC	0.97053	-0.24099	0.3362	0.016	*
NH_4^+-N	-0.69100	0.72285	0.1673	0.129	
NO_3^--N	-0.87666	0.48112	0.3883	0.009	**
AP	0.96586	-0.25905	0.3849	0.009	**
AK	0.94717	-0.32074	0.0526	0.587	
TN	0.92053	-0.39066	0.5294	0.001	***
TP	0.79300	0.60922	0.0944	0.364	
TK	-0.99541	0.09573	0.4527	0.001	***

Note: Significant codes: 0 “***”, 0.001 “**”, 0.01 “*”, 0.05 “.” 0.1 “ “, 1

Permutation: Free

Number of Permutations: 999

Table S5. The bacterial ASVs correlation between soil enzyme activities in different CC soil of patchouli.

***VECTORS					
	**ASV000001**	**ASV000002**	**r^2**	**Pr (> r)**	
ß-GC	-0.71852	0.69551	0.0471	0.648	
PPO	0.99518	-0.09803	0.2383	0.046	*
CAT	0.92948	0.36887	0.0841	0.392	
NAG	0.32626	0.94528	0.0079	0.926	
LAP	0.35517	0.93480	0.0231	0.794	

Note: Significant codes: 0 “***”, 0.001 “**”, 0.01 “*”, 0.05 “.” 0.1 “ “, 1

Permutation: Free

Number of Permutations: 999
